# Transformations in Diabetes Care: Lessons From Commons‐Based, Peer‐Produced Citizen Science

**DOI:** 10.1111/1467-9566.70140

**Published:** 2026-01-22

**Authors:** Shane O'Donnell, Muireann Quigley

**Affiliations:** ^1^ College of Business University College Dublin Dublin Ireland; ^2^ Birmingham Law School University of Birmingham Birmingham UK

**Keywords:** commoning, citizen science, diabetes, open source, user‐led innovation

## Abstract

Medical technologies have historically been the domain of private entities and public institutions. However, this market–state model is being challenged by commons‐based, peer‐produced citizen science. Here, we present a community of people with diabetes, which has developed life‐critical medical devices through open‐source innovation, as a case study in the possibilities and constraints of a more commons‐centric form of healthcare. We draw on insights from an existing body of social scientific research, as well as the first author's personal experience as a member of this movement, putting them in the context of a theoretical approach in which commoning is viewed as a transformative social paradigm. Drawing on Bollier and Helfrich's patterns of commoning, we offer two linked conceptual contributions which function as an analytical lens. First, we outline what we call the ‘diabetes commons’ to understand the dynamics of how the community established an alternative innovation ecosystem. Second, we posit ‘medical technology provisioning’, whereby production, consumption and follow‐up care are blended into a holistic whole. We highlight how promoting an open innovation model allowed the community to avoid enclosure and transformed the diabetes device landscape. We also argue that this openness has created challenges for its autonomy and sustainability as a commons.

## 1 Introduction

Until recently, the development, production and distribution of modern medical technologies have almost exclusively been the domain of either private entities or public institutions (Faulkner 2008; Abbott 2018, 29). Within this dominant paradigm, scientific knowledge has traditionally been ring‐fenced or enclosed through the enforcement of proprietary mechanisms, such as modern patent law or trade secrets (Bicudo et al. 2022). This approach is justified based on a particular worldview which holds that the enclosure of knowledge and the price incentives of the market are fundamental to incentivising innovation and on‐going engagement in research and development. This configuration of market/state innovation has been associated with a multiplicity of failures to adequately meet the needs of different stakeholders within the healthcare ecosystem. One such failure is the relatively slow diffusion of innovation that could be potentially life enhancing for people living with chronic conditions (Flowers 2017). The logic underpinning this dominant approach, however, is increasingly being challenged by self‐organised communities of people affected by different chronic conditions who are co‐creating their own medical technologies outside of standard pathways of device innovation and regulation (Flowers 2017; Kempner and Bailey 2019). One prominent example of this, and the focus of this article, is the so‐called #WeAreNotWaiting movement: a community of people living with diabetes who modify existing diabetes management devices through open‐source innovation. Over the last decade or so of its existence, members of this movement have created a safe and effective set of life‐critical medical technologies, known as open‐source automated insulin delivery (OS AID) systems, for the management of diabetes. Significantly, whereas the traditional proprietary approach of industry innovation involves fencing off and appropriating scientific knowledge, the #WeAreNotWaiting movement has achieved its technological advances through openness, collaboration and cooperation. Understanding how this alternative innovation ecosystem operates requires a conceptual lens that can account for collective practices of governance, sharing and care beyond market–state logics.

We address this by offering two linked contributions which extend commons theory into healthcare, something largely missing from existing literatures. First, we outline and develop the concept of the ‘diabetes commons’, situating this emergent user‐led commons within a small but growing body of literature that frames commoning and bottom up citizen science as a transformational social paradigm (Mahr and Dickel 2019; Bodini et al. 2021). Unlike most other user‐led commons examined in literature to date, the #WeAreNotWaiting community has moved from the periphery of the healthcare system to become a global movement, with tens of thousands of users worldwide. Therefore, it represents a unique case study in which to examine the affordances and constraints of commoning in contemporary healthcare and to critically examine its transformational potential. Second, building on Bollier and Helfrich's language of provisioning (2019), we introduce the concept of ‘medical technology provisioning’ to describe how members of the diabetes commons collectively maintain, adapt and share life‐sustaining technologies outside of market or institutional channels. In doing so, we build on but also go beyond existing work on commoning and citizen science. Although current literatures tend to focus on interpersonal support or distributed innovation, our framework conceptualises how these practices can coalesce into a shared system of stewardship and access through peer‐governance. Here, diabetes is particularly revealing because everyday survival depends on highly technical devices, thus making visible forms of patient‐led provisioning that usually remain hidden in other healthcare contexts. This case therefore lets us extend commons theory into healthcare while also offering a new way to think about how medical technologies can be provided and sustained. Although we do not position this paper as a piece of formal ethnographic or autoethnographic work, we acknowledge that its content and framing are necessarily informed by the first author's personal observations and experience as a member of this movement. Our contention is that whilst the #WeAreNotWaiting movement has led to significant transformations in diabetes care through bringing about a more commons‐centric form of healthcare, they also reveal some of the risks and constraints of such an approach.

## 2 Doing It for Themselves: The #WeAreNotWaiting Movement

Type 1 diabetes (T1D) is an autoimmune condition in which the insulin‐producing islets cells in the pancreas cease producing (or produce very little) endogenous insulin. Standardised treatment involves what is referred to as intensive insulin therapy, which usually takes the form of multiple daily injections self‐delivered via an insulin pen and routine monitoring of glucose levels through finger prick blood testing. In recent years, two technologies—the continuous glucose monitor (CGM) and the insulin pump—have begun to transform T1D management. A CGM measures the glucose levels in the fluid around a person's cells using a small sensor inserted just under the skin. The sensor is attached to a transmitter sitting on top of the skin which sends glucose readings to a display device, offering an almost complete picture of glucose trends in near real time. Insulin pumps are programmable devices which continuously deliver insulin using a small cannula inserted into the subcutaneous tissue. Despite the very real advantages of both CGM and insulin pump therapy, neither of these technologies on its own relieves the user of the need to make dozens of potentially life‐and‐death treatment decisions every day (Diabetes UK 2021). Each treatment decision still carries with it the risk of taking too much insulin, which can be lethal. This is why T1D is often referred to as the thinking person's disease as those living with the condition need to ‘calculate, adjust and constantly think about diabetes in order to survive’ (Mialet 2022, 106).

Exhausted by the demands of standard therapies, many people with diabetes (PwD) have looked to technological solutions to help to alleviate some of the emotional and cognitive burdens of living with the condition. However, commercially developed technological solutions for the management of diabetes have been perceived by many PwD to be too blunt. They are inadequate not only to the therapeutic tasks at hand—managing diabetes—but also to getting on with the business of living life in as unencumbered a manner as possible (Cleal et al. 2021). There has been particular frustration at the way in which diabetes technology companies were (and still are) using a proprietary model to develop devices such as CGMs and insulin pumps, whereby data is locked into single devices and not shareable across different platforms. Thus PwD (and those that care for them) often lack full access to, and control over, real‐time data that is essential to their self‐care (Reed‐Berendt et al. 2025). Given this, tech‐savvy individuals affected by diabetes began experimenting with commercial hardware to unpick the proprietary wall (Jansky and Langstrup 2022). Pooling their collective efforts, members of the diabetes community developed open source software called ‘Nightscout’ that enabled users to send their CGM data to the cloud where it could be accessed by any device of their choosing—effectively creating the world's first remote monitoring system for people with diabetes (Desborough 2023).

These developments in turn opened the door to even more ambitious forms of horizontal and peer‐to‐peer forms of innovation. The most significant of these came in 2013 when Dana Lewis and her partner Scott Leibrand, in collaboration with others within the community, were able to develop a programme that ‘closes the loop’ between CGM and insulin pump and delegates insulin dosing decisions to an algorithm. After careful experimentation and refining the algorithm, Lewis and Leibrand made the source code publicly available through open source channels so that others could build it for themselves (Lewis 2019). With the efforts of commercial players in developing equivalent technology lagging behind by several years, open source automated insulin delivery (OS AID) systems and Nightscout were quickly taken up by PwD across multiple countries. Coalescing under the banner #WeAreNotWaiting, this movement consists now of over 10,000 users worldwide (Braune et al. 2022).

There are currently five main open‐source AID systems: OpenAPS, AndroidAPS, Loop, iAPS and Trio. In the most basic implementation of all five, the algorithm makes incremental adjustments to basal (background) insulin delivery in response to projected glucose levels while also taking into account certain parameters set by the individual user. These systems are comprised of open‐source software installed either on a smartphone or a minicomputer, a CGM and an insulin pump. The software allows a person's CGM and insulin pump to ‘talk’ to each other, enabling insulin doses to be adjusted and delivered automatically in near real‐time. These systems are ‘do‐it‐yourself’ (DIY) in the sense that users have to compile and maintain the code themselves. However, a whole community of experienced users are available to assist those less IT confident users on their journey of building and maintaining the software (Crocket 2020). A growing body of evidence has emerged showing that OS AID can lead to significant improvements in self‐reported clinical outcomes and in quality of life, and that these systems can have a transformational impact on the lived experiences of end users (Braune et al. 2021). Moreover, their safety and effectiveness have also recently been corroborated in randomised controlled trials (Gawrecki et al. 2021; Burnside et al. 2022).

## 3 The Making of the Diabetes Commons

Having outlined the history and development of the #WeAreNotWaiting movement, in this section, we introduce and analyse what we are calling the ‘diabetes commons’. Our analysis is situated at the intersection of citizen science and commons theory. It focuses on how members of the #WeAreNotWaiting movement enact a commons‐centric model of healthcare, operationalised through medical technology provisioning, in order to better meet their own healthcare needs.

### 3.1 Towards a Reimagined Commons‐Based Citizen Science?

‘Citizen science’ is broadly used to refer to the involvement of nonprofessional citizen participants at various stages of the scientific research process, including in the biomedical sphere (Wiggins and Wilbanks 2019). This has predominantly focused on areas such as data collection and genomics such as genetic sequencing or working towards creating biomarker‐based tests for rare diseases (Radu et al. 2021). However, as Mahr and Dickel (2019) point out most projects labelled as citizen science take a top‐down approach through which citizens are engaged not as equal actors but as ‘invited contributors’, with decision making remaining firmly in the hands of professional scientists. Therefore, despite the democratic qualities that the name seems to invoke, dominant approaches to citizen science may inadvertently reinforce traditional power structures and the divide between ‘experts’ and ‘laypersons’ (Mahr and Dickel 2019). In contrast to top–down approaches to citizen science, we have seen the emergence of citizen collectives with their own selfgoverned infrastructures in the form of DIY communities, maker movements and user‐led innovations (Mahr and Dickel 2019; Pelacho et al. 2021). Here, commons theory can help us to further elucidate the transformative potential of these more autonomous forms of citizen science.

Contemporary commons scholarship owes much to Elinor Ostrom who, in a direct refutation of the prevailing myth of the ‘tragedy of the commons’, demonstrated empirically how resources can be managed sustainability by a community without the need for either external regulation or privatisation (Ostrom 2015). Although approaching the subject with an economist's lens, her insights have spawned a vast literature that provides a sociologically rich understanding of the commons. Out of this has emerged something of a consensus that a commons can be understood as a community that comes together to co‐create, care for and enhance a resource according to its own rules and governance structures (Bollier and Helfrich 2019). Importantly, a resource is not a commons in and of itself (e.g., oceans or the atmosphere). Hence the verb to ‘common’ or ‘commoning’ is used to underline that a commons only comes into being through the on‐going social practices of a community (Bollier and Helfrich 2019).

A more recent body of literature has sought to depart from Ostrom's economistic approach by theorising how the commons emerge and operate in the political–economic context of market capitalism and in relation to the state (Wagenaar and Bartels 2024, 338). From this point of view, the commons is conceptualised not only as a regime for the management of resources but also as a mode of social organisation that has the potential to transcend the current neoliberal political economy (Bauwens et al. 2019; Linebaugh 2014; Papadimitropoulos 2020; Bollier and Helfrich 2019). For Bollier and Helfrich (2019) the commons can be understood as both a new and old social paradigm as people have been meeting their needs through the practice of commoning for millennia. Historically, the commons have been restricted to small group dynamics in highly localised settings, such as forests, fisheries, pastures, etc. However, according to Bauwens et al. (2019), the peer‐to‐peer relations engendered by the internet enabled what they describe as new ‘nonterritorially situated’ forms of self‐organisation. This in turn has created historically unprecedented affordances for the massive scaling up of commons dynamics. A notable example is in the realm of the digital commons where peers co‐create resources through distributed social networks rather than the traditional hierarchical arrangements of public or private institutions (Benkler 2006). Open‐source communities have created artefacts that are in many cases vastly superior to their commercial equivalents (e.g., Wikipedia and free and open‐source software) and upended sectors of the economy where proprietary business models traditionally dominated (Benkler 2006).

Given this capacity for subverting existing paradigms and practices, some STS scholars have pointed to the transformational potential of commons‐based citizen science in healthcare (Morell et al. 2021; Mahr and Dickel 2019). Mahr and Dickel, for example, have argued that DIY communities may usher in an epistemic revolution ‘that might even influence the scientific mainstream and reshape and renegotiate the boundaries of knowledge production—both in terms of inherent power structures and of the relation of science to other societal sub‐systems like health care and economy’ (Mahr and Dickel 2019, 16). However, most self‐organised communities that are cited in the literature have, for a variety of reasons, been unable to move from the margins to mainstream healthcare. In contrast, the substantial influence that the #WeAreNotWaiting movement has had on the diabetes care landscape presents an opportunity to examine the transformative potential of commons and commoning in healthcare.

### 3.2 Medical Technology Provisioning and the Commoning of Diabetes

Like most medical technologies, the algorithms underlying commercial AID systems typically (though by no means exclusively) originate through research undertaken at institutions such as universities and are then brought to market by profit‐driven entities. Because these commercial AID systems are co‐created by more than one entity, this necessitates a range of commercial, intellectual and technical agreements to be put in place between the parties. Additionally, there needs to be agreement on design controls, quality controls, responsible parties, etc. The result is that it can take over 8 years and 10s of millions of dollars for a commercial AID to be brought to market (Desborough, 2023). By contrast, OS AID systems are developed under a completely different logic. The primary goal is not to bring innovations to market but to meet the shared needs of a community of PwD (Bollier and Helfrich 2019, 166–67). The use value of the innovation, rather than its exchange value in the marketplace, is thus a key element that separates open source and commercial AID. Software developers within the community are typically either end‐users themselves or care for someone who is an end‐user. Thus, the usual boundaries between production and consumption are blurred to the point where the development, distribution and subsequent use of OS AID (and other community resources) are blended into an integrated whole.

The collapsing of these boundaries and (re)integration of production and consumption within a commons is referred to by Bollier and Helfrich as ‘provisioning’ (Bollier and Helfrich 2019, 81). In contrast to the traditional paradigm in pharmaceutical and medical technology sectors where there is strict separation between production and consumption, a basic goal of provisioning in a commons is to ‘reintegrate economic behaviours with the rest of one's life, including social well‐being, ecological relationships and ethical concerns’ (Bollier and Helfrich 2019, 87). In what follows, we focus on four interrelated patterns and behaviours that the #WeAreNotWaiting community enacts in the ‘provisioning’ of their innovations: sharing knowledge generously, the use of convivial tools, lack of reliance on hierarchy, and supporting care and de‐commodified work.

The foundational pattern within #WeAreNotWaiting is that members of the community contribute and share knowledge (ideas, codes, designs and mentorship) generously (Bollier and Helfrich 2019). The community is made possible by ‘gentle reciprocity’ which characterises the social life of the movement (Bollier and Helfrich 2019, 74), that is, the willingness to give, without the expectation to receive anything in return, except for the hope of reducing the burden of diabetes for oneself and others. This ethic of contributing and sharing is encapsulated in the community mantra of ‘pay‐it‐forward’ (Geiger and Stendahl 2024, 2524) and is exemplified in Lewis and Leibrand's decision to waive any proprietary rights to their original AID system. Open sourcing of the software licence made subsequent versions of OS AID such as AndroidAPS and Trio possible. In this way, OS AID systems are also an example of what Bollier and Helfrich describe as convivial tools and technologies; they are ‘open‐ended systems that anyone can use and adapt for their own purposes’ (Bollier and Helfrich 2019, 190).

Convivial tools stand in contrast to the closed systems that characterise most commercial medical devices. Commercial devices tend to lock users into particular ways of using the device. Indeed, prior to the emergence of Nightscout, the only way for (carers of) PwD to access, visualise and interact with blood glucose values was to do so via the manufacturers' own devices and interfaces (Lewis 2019). However, the manufacturers' proprietary systems lacked functionalities—such as remote monitoring capability—viewed by many PwD as essential to their own self‐care. Additionally, commercial devices prohibit inspection of, amongst other things, the source code of device software without the explicit permission of the manufacturer. Users can neither interrogate the software/algorithms on their devices nor access their own data generated by them. Consequently, users (and healthcare professionals) have little understanding of how commercial AID systems work because the knowledge behind it is enclosed through proprietary trade secrets. Conversely, by building AID systems that are open, accessible, modifiable and sharable, the #WeAreNotWaiting community enables users of its tools to more easily adapt the technology to their individual needs. Moreover, every facet of how the algorithms operate is public knowledge, allowing anyone to audit and evaluate the code and make suggested improvements (Braune et al. 2023). In short, convivial tools enable the community to collectively determine the development of the technology that they rely on as a matter of life and death.

In the development and making available of these convivial tools and innovations, the #WeAreNotWaiting community relies on informal peer governance to guide its activities. In this regard, the #WeAreNotWaiting movement can be seen as an example of a heterarchy, meaning different types of rules and organisational structures are deployed depending on the circumstances or task at hand. This includes, for example, top–down hierarchies and bottom–up participation (both of which are vertical), as well as peer‐to‐peer dynamics (which are horizontal) (Bollier and Helfrich 2019, 82). On one level, the entire innovation ecosystem is based on what Bauwens and colleagues (2019) refers to as permissionless contributions. Each individual member of the community is free to bring their own situated knowledge of diabetes to tinker with and improve the code (Jansky 2023). However, it is also common practice that the results of those experiments will be fed back to the community. Mirroring the ‘collective self‐experimentation’ described elsewhere by Kempner and Bailey (2019, 3), it is this continuous feedback loop between the individual self‐experimenter and the collective intelligence of the wider community that drives advancements in OS AID algorithms (Kempner and Bailey 2019; Jansky 2023). By side‐stepping the usual time consuming and onerous processes involved in medical device development (including navigating and meeting a variety of regulatory requirements), users can therefore effect a relatively fast innovation cycle.

However, although bottom–up participation and peer‐to‐peer sharing means that everyone is free to contribute and suggest changes to the code, a final decision on whether the modified code *should* be incorporated into the main version are usually taken on review by a smaller group of peers (or even solely by one person). However, the power to control the direction of software development is not absolute. An example of this can be found in the case of iAPS which up until recently had been one of the most popular OS AID systems. Here, users of iAPS were concerned that the software's development had become too centralised by the lead developer and increasingly lacked meaningful input others within the community. According to this group, the latest updates of iAPS were being pushed to the main version without sufficient prior testing and peer review before general release, potentially compromising the safety of the system. Consequently, a group of around 50 volunteers decided to create a new software project called Trio, which would use some of the original source code of iAPS, but which would be developed in such a way as to meet the standards of open source development (Eastzer 2024b). The case of iAPS/Trio shows that while hierarchies do exist within the community, they are not necessarily as fixed nor as rigid as would be the case in commercial settings. It also shows that the diabetes commons can be both self‐regulating and fast‐acting when it comes to ensuring the safety of its innovations.

Beyond the development of the technologies and tools themselves, integral to the diabetes commons is a fundamentally different type of care. As a result of developing their innovation outside of formalised healthcare settings, the #WeAreNotWaiting community has created its own informal care systems (Jansky 2023), something which exemplifies the type of decommodified work that is more typically found within a commons (Bollier and Helfrich 2019). This care and support takes a variety of forms, including, in‐person ‘build sessions’ where participants can learn how to build such systems in the presence of a more experienced member of the community, and online fora where continuing support and help is available. Of particular note here is Braune and colleagues' findings (2021) that OS AID users often deem the care they experience as part of the community superior to that typically provided by traditional device manufacturers and health care professionals. Although such sentiments were not necessarily shared by all participants in their study, they bring to mind Mol's assertion that market mediated forms of care are, by their nature, limited to the quid pro quo of the transaction at hand (Mol 2008). In this sense, the care on offer by commercial companies must be pre‐defined with a clear beginning, middle and end. By contrast, the diabetes commons offers a more open‐ended form of care (Mol 2008).

The boundaries of care, much like the boundaries between production and consumption, are also blurred within the process of medical technology provisioning. By this we mean that within the diabetes commons, there is no separation between the technical, clinical, psychological, etc. The care received by community members goes beyond support in building and maintaining the software but also encompasses the integration of self‐care technology into one's life in a way that leads to reduced disease burden. Arguably, this more holistic form of care is potentially more well equipped to support people in navigating the vagaries of living with a chronic condition than that which can be typically provided in formal care settings. Finally, it is important to note this form of care also generates dynamics that are not typified by consumer behaviour (Mol 2008, 80). For example, community members who learn to build a system with the support of more tech‐savvy community members often ‘pay‐it‐forward’ through contributing skills in other areas such as documentation writing or the translation of documentation. Thus the care provided through the diabetes commons is often an ongoing interaction in which ‘in one moment you care, and in the next you are taken care of’ (Mol 2008, 80). In such a way, the culture of gentle reciprocity extends well beyond contributing and sharing in the ongoing development of software. It produces a virtuous cycle which enables the community to reproduce itself, thus not only making but also maintaining and extending the diabetes commons.

## 4 Transformations and Challenges

Having examined some of the key internal dynamics relating to a particular type of medical technology provisioning, we note that the stability and success of any given commons is also shaped by its external relationships with the market and state (Bollier and Helfrich 2019). Historically, both market and state institutions have sought to ring‐fence and enclose the commons through privatisation, commodification and/or prohibiting the practice of commoning through legal mechanisms (Linebaugh 2014). Given this, it might have been expected that, as the #WeAreNotWaiting movement grew in terms of size and influence, the response of dominant ecosystem players would have been to find legally enforceable ways of shutting the movement down. Initially, this is what happened (see for example Moody 2020; FDA 2019). Remarkably, however, the #WeAreNotWaiting movement survived these threats of enclosure and, as we are about to see, in the process re‐shaped market and state dynamics in diabetes care. However, despite the relative success of the diabetes commons, it faces ongoing challenges which mean that the future of the movement is far from secure.

### 4.1 Three Spheres of Transformation

The relative success of the #WeAreNotWaiting movement can in part be explained by its ability to support a large number people with diabetes to ‘take the means of production into their own hands and create the advocated change themselves’ (Jansky and Langstrup 2022, 508). The consequence of this is that a significant number of people stepped outside of the market–state nexus to choose alternative technologies and a different form of care. However, the pioneers of the #WeAreNotWaiting movement from the outset were also of the belief that an approach to dominant stakeholders characterised purely by hostility and antagonism would likely result in their activities quickly being shut down. Thus, they aimed to reorientate the relationship so that it was defined by collaboration and openness. By presenting their innovations as being complementary, rather than in opposition, to industry and mainstream medicine (Demonaco et al. 2020), they were able to create and advance an alternative vision of the future of diabetes care. In this, different actors could all work together to improve and hasten the development of diabetes technologies through improving innovation and regulatory pathways. This form of ‘device activism’ as Jansky and Langstrup (2022) have describe it resulted in tangible transformations on a number of fronts.

The first of these is healthcare practice. OS AID users increasingly came to speak on their experiences via online social fora and via private conversations with their healthcare professionals in clinic. These conversations, combined with the growing evidence of safety and efficacy of OS AID, have led to shifting attitudes among some healthcare professionals in formal healthcare settings (Davis 2024). Indeed, moderators of #WeAreNotWaiting forums such as Loop and Learn, have reported an increasing number of users who have come seeking support of the community based on a recommendation made by their healthcare professionals (J. Milo, personal communication, 03 December 2024). Some clinics have even begun to support in‐house set up of OS AID. In Canada, BCDiabetes, a privately operated and publicly funded online clinic offers ‘clinic‐run, clinic‐provided’ OS AID systems to users in Canada. This ‘supported open‐source automated insulin delivery’ (SOS AID), as they refer to it, is preferred over retail AID systems. As noted by Samuel et al. (2024) because Canadian insurance companies currently only offer partial coverage for commercial AID components, it is a more affordable option to people with diabetes. In addition, the Steno Diabetes Centre, one of the largest clinics dedicated to diabetes in the Nordic countries, has hosted several so called ‘build parties’ where experienced members of the #WeAreNotWaiting community demonstrate how to build OS AID to newcomers (L. Raimond, personal communication 24 November 2024). These examples are significant because they demonstrate a certain willingness of stakeholders in formal healthcare settings to engage with and support the diabetes commons.

Even in clinics where OS AID has not been officially endorsed and adopted, the impact of the #WeAreNotWaiting movement is evident. The superior nature of the technological advancements developed by the community served as an indictment of the existing system. This, combined with PwD choosing to adopt OS solutions, forced dominant stakeholders such as the U.S. Food and Drug Administration (FDA) and device manufactures to take the needs of the community more seriously (Geiger and Stendahl 2024, 2513–2415). The pressure exerted by the #WeAreNotWaiting movement has culminated in recent significant changes to healthcare policy. In the UK, for example, the National Institute for Health and Care Excellence has recently recommended the use of hybrid closed loop systems for the effective management of type 1 diabetes (NICE 2023) and NHS England is developing a 5‐year plan for phased uptake of the technology (NICE 2023, para 4.4). Although this guidance and the technology appraisal underpinning it relates to approved and available commercial systems, without the work of the #WeAreNotWaiting community, we might still be waiting for these to be developed. Moreover, the alliances forged with clinicians and other healthcare professionals, and the advocacy work of the community in general, has undoubtedly helped to focus attention of health policymakers on the needs of those with T1D.

Second, the transformative potential of the diabetes commons is also being felt in the regulatory sphere. Recently Tidepool Loop, an AID app that was partly based on the open‐source algorithm known as Loop, has gained regulatory clearance from the US FDA (Downey et al. 2023). This is ground‐breaking because it marks the first time that an app, which began life as a user designed and developed technology, has been shepherded through the regulatory process in the United States or any other jurisdiction. It is also unique in that it is designed to be interoperable with other AID device components, meaning that (in theory at least) it could be compatible with any number of commercially developed CGMs and insulin pumps (Braune et al. 2023; Downey et al. 2023). The wider significance of this lies in the effect on what we might term the regulatory imagination. Although as little as 5 years ago, it seemed highly unlikely (perhaps impossible) that regulatory approval would be given to a medical device with its origins in a user‐led open source movement, the success of Tidepool in navigating the requirements of the regulatory complex in the United States opens up the possibility that something similar could be achieved in other jurisdictions not only by Tidepool but also potentially by other open innovation initiatives (Downey et al. 2023).

Third is the impact of the diabetes commons on industry. There is by now a widespread acknowledgement, even among industry insiders, that the pressure exerted by the #WeAreNotWaiting movement on key stakeholders played a significant role in forcing incumbent device vendors to bring AID technology to market far sooner than they had anticipated (Geiger and Stendahl 2024). Since 2015, several commercial hybrid closed loop systems are now available for clinicians to prescribe to healthcare users (Kesavadev et al. 2020). Arguably, however, the fruits of the drive for openness and cross‐fertilisation can be better seen with regards to newer entrants to the commercial AID market, some of which are actively incorporating innovations initially developed by the community as part of their product pipeline. Perhaps the most significant of these is the recent FDA clearance of the insulin pump Twiist (DiaTribe 2025). Twiist is significant because it is the first insulin pump to integrate with the Tidepool Loop algorithm (Eastzer 2024). The CEO of Sequal, the company behind Twiist, is on the record stating that integrating with Tidepool Loop eliminated the need to develop their own hybrid closed‐loop algorithm in‐house and thus helped reduce the complexity of bringing their product to market (Eastzer 2024). These developments show how open source innovation can assist new players to compete in a device market which has historically been concentrated in the hands of a few dominant firms. As such, through the practice of commoning, the community helped to reshape industry in a way that would likely have been highly improbable had they engaged soley in more traditional forms of patient activism that involve petitioning of state actors (Bollier and Helfrich 2019).

### 4.2 Challenges Within and Beyond the Diabetes Commons

The success of the #WeAreNotWaiting movement in concretely influencing commercial innovation has led it to being celebrated as a leading example of user‐led innovation (Demonaco et al. 2020). However, more recent developments have shown limitations in how far incumbent device manufacturers are willing to go in order to accommodate the #WeAreNotWaiting movement. Many pioneers within the community hoped for a future in which there would be greater complementarity between user‐driven innovation and the commercial medical innovation systems. In this vision, device manufacturers would benefit from availing of free research and development by the community which in turn, would encourage them to make their interfaces open (Demonaco et al. 2020, 87). Yet this promissory vision belies the ways in which a position of openness has so far failed to fully transform industry practices in a way that had been hoped/expected by the community. This is reflected in established device manufacturers ultimately opting out of the interoperable ecosystem proposed as part of the Tidepool Loop initiative and instead prioritising what might be considered a more traditional proprietary model (Look 2023). Thus, the broader vision of the #WeAreNotWaiting pioneers' of an open model of innovation becoming standard practice within industry and healthcare perhaps seems as far away as ever.

Moreover, although open‐source innovation in diabetes care has been tolerated by industry in so far as it provides a new source of value extraction, neither market (nor state) actors have reciprocated by contributing back to the community. Despite pursuing a strategy of openness and partnership for almost a decade, and for all the contributions #WeAreNotWaiting members have made to advancing the field of diabetes technology, the community remains under threat of enclosure. For example, the movement relies on full access to the real‐time data generated by diabetes devices in order for their own applications (e.g., Nightscout and the different OS AID systems) to work. However, device vendors, citing cybersecurity concerns and the need to safeguard user data, are implementing increasingly restrictive data governance policies (Desborough 2023). As the security protocols of the device vendors increase in sophistication, there is a risk that reverse engineering will become too resource and time intensive for developers within the community, a situation which would put the entire open‐source ecosystem in jeopardy (Desborough 2023).

All of this has significant consequences for the community in managing itself as a commons. Indeed, as the numbers of people who use OS AID innovations continues to grow, so too has demand for a core team of software maintainers, moderators, and documentation writers, some of whom have left to work for industry while others have reported burnout (Jansky 2023). In the absence of any compensation for their contributions, community members must constantly manage the tension between finding the time and energy to contribute to the community and the need to earn a livelihood (Gerhardt 2023). With the community increasingly starved of resources it needs to sustain itself, some longstanding pioneers are advocating further commercialisation as a solution. For example, pay‐for‐service versions of Nightscout are now being offered to users who do not have the time or IT confidence to build the software themselves, with the profits enabling the hiring of a team who can dedicate themselves to the on‐going maintenance of the Nightscout source code. However, this has created a schism with others within the community who have argued it undermines the ethos of indirect reciprocity (pay‐it‐forward) that has been central to the movement's success (Desborough 2023).

Somewhat paradoxically, even as the #WeAreNotWaiting movement has managed to move from the fringes to the mainstream, its survival as a commons has never looked more precarious. Thus, continued evolution and alternative iterations of this particular commons‐centric model are likely needed in order to ensure further and lasting transformations in diabetes care and beyond. Interestingly, other user‐led initiatives are under way which may at least partially go some way towards addressing this. Inspired by the case of Tidepool Loop and the #WeAreNotWaiing movement more generally, a number of different projects are exploring how an open source approach might be applied to hardware production and distribution (Payne, Pooke, Holder‐Pearson, et al. 2024; Payne, Pooke, Wilkinson, et al. 2024; Karia et al. 2019). For example, motivated by the stark inequalities in access to diabetes care in Māori, Pacific and Indo‐Asian ethnic populations, efforts are underway by a New Zealand‐based research group to develop an ultra‐low‐cost 3D printed insulin pump which runs on open‐source software (Payne et al. 2022). The pump has already undergone bench safety and first in human feasibility testing (Payne, Pooke, Holder‐Pearson, et al. 2024; Payne, Pooke, Wilkinson, et al. 2024). This collaboration between biomedical researchers and members of the diabetes community envisions using a distributed model of manufacturing. Here partnerships with generic manufactures or not‐for‐profit entities would be established and the 3D printed pumps would be sold at near production cost. By focussing on ultra‐low‐cost production, this distributed model would have potential to bring much needed diabetes technology to low‐resource settings. Potentially, the entry of an open source insulin pump into the market would also make the community at least somewhat less reliant on the whims of incumbent device manufacturers who up to this point have remained wedded to a proprietary approach.

## 5 Discussion

Social and technological trends are increasingly providing affordances for self‐organised communities living with different chronic conditions to produce their own medical devices and treatments, thereby challenging the dominance of the market/state model. The activities of the #WeAreNotWaiting movement share several characteristics with commons‐based citizen science. They are a self‐funded, self‐taught, open‐source community who, through bottom–up forms of citizen science, have created new software and developed advanced expertise in diabetes technology outside of traditional clinical and industry‐led medical device environments. As we have argued in this article, OS AID represents a critical empirical case study of user‐led commons in a healthcare context. By introducing the concepts of the diabetes commons and medical technology provisioning, we extend commons theory into a domain where life‐sustaining technologies are typically governed by market–state logics. This framework foregrounds how practices of maintenance, adaptation and peer‐to‐peer sharing constitute an alternative provisioning system that challenges assumptions about how medical technologies can and should be developed, distributed and cared for. The community enacts patterns and behaviours of commoning that have enabled it to establish itself as an alternative to the commercial development, production and distribution of a particular type of medical technology (automated insulin delivery) for a large community of users.

Central to the success of the movement as a commons has been the blending of production, implementation, consumption and follow‐up care through medical technology provisioning. It has eschewed the traditional proprietary approach of industry, involving the fencing off and appropriation of scientific knowledge, as well as a lack of transparency and openness with respect to the development of diabetes technologies. Instead, the community has developed an alternative innovation ecosystem that is based on open‐source knowledge sharing of ideas, codes, designs, mentorship, developing convivial tools, and which relies on peer governance to guide its activities. It has also built informal systems of care that are, as far as many users are concerned, superior to professional support available in formalised healthcare settings. Therefore, the infrastructures of innovation and care generated by the #WeAreNotWaiting community offer insights into how commoning can help transcend the limitations of current market mediated forms of care. In addition, the impact of medical technology provisioning has gone beyond the borders of the diabetes commons (and the lives of those who use its innovations). It has substantively influenced and transformed mainstream medico‐scientific, healthcare and regulatory landscapes. However, we have also seen that the community's longstanding adoption of openness vis‐à‐vis industry, while critical to its success, has also created dilemmas which have the potential to overwhelm the community. Our analysis of the #WeAreNotWaiting community thus adds to literature by providing insight into what a more commons centric form of healthcare might look like, as well as the challenges of bringing such a model about.

It is necessary to acknowledge a number of limitations in our approach. First, inherent in our analysis is the normative claim that the diabetes commons contributes to the common good in healthcare (Geiger 2021). However, there are features of open‐source patient‐led innovation which could arguably be said to run contrary to this claim, not least when it comes the question of equity. Indeed, a number of studies have brought attention to the ways in which members of the #WeAreNotWaiting movement tend to be embedded within relatively privileged socioeconomic spaces, usually in the Global North (Gottlieb 2021; Jansky et al. 2024). Viewed in this way, some have (not unreasonably) concluded that patient‐led open‐source innovation may inadvertently have the potential to contribute to widening of health inequalities (Jansky et al. 2024). However, by narrowly focussing on the socio‐demographics of its user base, it is easy to overlook how the ‘device activism’ of the #WeAreNotWaiting movement has, as we have argued, changed the diabetes landscape in ways that have consequences for access to advanced diabetes technology across the population. As noted, market–state players have been forced to hasten the availability of commercial offerings as a result of the disruptive impact of the #WeAreNotWaiting movement. This is likely to result in AID technology diffusing faster amongst the broader population, particularly in advanced welfare state countries such as the UK. Such ‘spillover effects’ are one example of many potential ways in which PwD may benefit from the existence of the diabetes commons, regardless of whether or not they are active participants. Yet, another example is how the #WeAreNotWaiting movement has inspired novel (open source) approaches to making diabetes hardware more affordable. Although still in their infancy, these initiatives may provide the embryonic forms of an alternative to the current market–state model which is arguably structurally incapable of providing affordable and accessible advanced technology to the majority of the world's population. All of which is to say is that it is highly difficult to predict with any degree of certainty what the longer term impact of the #WeAreNotWaiting movement will be on inequalities in diabetes outcomes. However, a more commons centric form of healthcare could have the potential to move us much closer towards achieving the goal of making life enhancing diabetes technologies available for everyone who needs them.

Another limitation of the current study is that it has not analysed with adequate depth the gendered aspects of the #WeAreNotWaiting movement. However, along with the observations of other scholars, who have shown how care and participation are often not evenly shared (Mullins and Cooke 2015; Schneider 2022), we note that the majority of contributions on developing and maintaining the code for OS AID is carried out by men. Conversely, the vast majority of those engaged in what Mullins and Cooke (2015) describe as ‘noncoding contributors in open source’ (e.g., maintaining the forums, writing documentation and managing conflict) are women. In this sense, the dynamics of the community may be viewed as mirroring the feminised labour forms seen in other areas of life where (often undervalued) emotional work is left to women (Schneider 2022). There is, therefore, a need for further research into what these (gendered) divisions of labour might mean for the sustainability of the movement. At the same time, it must also be noted that the #WeAreNotWaiting community has been at the forefront of drawing attention to gender inequities in the development of formal scientific knowledge surrounding diabetes. For example, members of the community have brought attention to the metabolic challenges associated with the menopause, an element of diabetes management that has up until recently has not recieved adaquate attention from credentialed experts (Hossmann et al. 2025).

Finally, it is also important to acknowledge that the particularities of the diabetes commons mean that some of the conditions critical to its success may not necessarily be readily emulated in full by communities (self‐)organised around other chronic conditions. For example, although the making and operation of the diabetes commons has resisted, and to a large extent functioned outside of, standard healthcare practices and institutions, its success has nevertheless only been made possible by its ability to convince the biomedical and scientific establishment of the legitimacy of its innovations. In this regard, having (open source) software (as opposed to either biological agents or traditional pharmaceuticals) as the focal point of their innovations proved to be an advantage to the #WeAreNotWaiting in several ways. For example, CGM data, combined with other data points from the insulin pump, allows the community to demonstrate the impact of the OS AID algorithm on an almost minute‐by‐minute basis, becoming ‘a source of collective epistemic power’ (Davis 2024, 629). This created the conditions for collaborations with researchers and the co‐creation of a formalised scientific evidence base (Burnside et al. 2022). Such technical infrastructure may not be available for other chronic communities engaging in similar practices (Flowers 2017). Further empirical analysis is therefore needed to examine whether such communities can move beyond the fringes of the ‘institutional boundaries of scientific knowledge production’ (Mahr and Dickel 2019, 16).

Despite the difficulties faced as the #WeAreNotWaiting movement moves into its second decade of existence, our study demonstrates how the commons can become a (re‐)emerging social force with the potential to reconfigure the dynamics of both market and state to bring them more into line with the needs of healthcare users. Moreover, by showing how the enclosure and privatisation of scientific and technical knowledge are not a prerequisite for the development of AID systems, the #WeAreNotWaiting movement has helped reimagine the potentials of the collective governance of health technologies. Quite apart from the technological innovations, the real legacy of the #WeAreNotWaiting movement may be that it has opened up new visions and possible futures regarding not only the development of commons‐based, peer‐produced technologies, but their distribution on a social benefit rather than for‐profit model. Therefore, it may prefigure new systems of healthcare provisioning that are more equipped to deal with contemporary challenges than those provided by (20th century) market–state models.

## Author Contributions

**Shane O'Donnell:** conceptualization, investigation, funding acquisition, writing – original draft, writing – review and editing. **Muireann Quigley:** conceptualization, investigation, funding acquisition, writing – original draft, writing – review and editing, supervision.

## Funding

Wellcome Trust (Grant No. 212507/Z/18/Z); the European Union (101064383) and Research England Quality‐related Funding.

## Ethics Statement

The authors have nothing to report.

## Conflicts of Interest

The authors declare no conflicts of interest.

## Data Availability

The authors have nothing to report.
